# Geographic miss of lung tumours due to respiratory motion: a comparison of 3D vs 4D PET/CT defined target volumes

**DOI:** 10.1186/s13014-014-0291-6

**Published:** 2014-12-16

**Authors:** Jason Callahan, Tomas Kron, Shankar Siva, Nathalie Simoens, Amanda Edgar, Sarah Everitt, Michal E Schneider, Rodney J Hicks

**Affiliations:** Department of Medical Imaging and Radiation Science, Monash University, East Melbourne, Victoria Australia; Peter MacCallum Cancer Centre, Centre for Molecular Imaging, St Andrews Place, East Melbourne, Victoria Australia; Sir Peter MacCallum Department of Oncology, The University of Melbourne, East Melbourne, Victoria Australia; Division of Radiation Oncology, Peter MacCallum Cancer Centre, East Melbourne, Victoria Australia; University of Nijmegen, Netherlands, Nijmegen, Netherlands

**Keywords:** 4D-PET/CT, Geographic miss, Lung cancer, Margins, Radiotherapy

## Abstract

**Background:**

PET/CT scans acquired in the radiotherapy treatment position are typically performed without compensating for respiratory motion. The purpose of this study was to investigate geographic miss of lung tumours due to respiratory motion for target volumes defined on a standard 3D-PET/CT.

**Methods:**

29 patients staged for pulmonary malignancy who completed both a 3D-PET/CT and 4D-PET/CT were included. A 3D-Gross Tumour Volume (GTV) was defined on the standard whole body PET/CT scan. Subsequently a 4D-GTV was defined on a 4D-PET/CT MIP. A 5 mm, 10 mm, 15 mm symmetrical and 15×10 mm asymmetrical Planning Target Volume (PTV) was created by expanding the 3D-GTV and 4D-GTV’s. A 3D conformal plan was generated and calculated to cover the 3D-PTV. The 3D plan was transferred to the 4D-PTV and analysed for geographic miss. Three types of miss were measured. Type 1: any part of the 4D-GTV outside the 3D-PTV. Type 2: any part of the 4D-PTV outside the 3D-PTV. Type 3: any part of the 4D-PTV receiving less than 95% of the prescribed dose. The lesion motion was measured to look at the association between lesion motion and geographic miss.

**Results:**

When a standard 15 mm or asymmetrical PTV margin was used there were 1/29 (3%) Type 1 misses. This increased 7/29 (24%) for the 10 mm margin and 23/29 (79%) for a 5 mm margin. All patients for all margins had a Type 2 geographic miss. There was a Type 3 miss in 25 out of 29 cases in the 5, 10, and 15 mm PTV margin groups. The asymmetrical margin had one additional Type 3 miss. Pearson analysis showed a correlation (p < 0.01) between lesion motion and the severity of the different types of geographic miss.

**Conclusion:**

Without any form of motion suppression, the current standard of a 3D- PET/CT and 15 mm PTV margin employed for lung lesions has an increasing risk of significant geographic miss when tumour motion increases. Use of smaller asymmetric margins in the cranio-caudal direction does not comprise tumour coverage. Reducing PTV margins for volumes defined on 3D-PET/CT will greatly increase the chance and severity of a geometric miss due to respiratory motion. 4D-imaging reduces the risk of geographic miss across the population of tumour sizes and magnitude of motion investigated in the study.

## Background

The use of integrated PET/CT in establishing radiotherapy target volumes is becoming increasingly accepted as the optimal approach for many malignancies, in particular lung cancer [1,2]. When defining target volumes for lung cancer the information obtained from the two imaging modalities provides complementary information about both tumour morphology (CT) and physiology (PET) [3]. This allows optimal definition of the tumour with good reproducibility while sparing delivery of dose to normal organs [4,5]. However a lung tumour can exhibit significant respiratory motion due to normal breathing, thus increasing the risk of geographic miss [6,7]. Therefore, target volumes need to be defined in order to minimize the potential of geographic miss during respiratory motion.

In many centres, PET/CT scans acquired in the radiotherapy treatment position is typically performed without compensating for the effects of respiratory motion. Areas most affected by respiratory motion are the lungs, liver and upper abdomen, particularly for structures close to the diaphragm. The standard method to account for motion is to apply a large margin to the gross tumour volume (GTV) to ensure adequate tumour coverage in a one-size fits all approach. However, a number of studies have found that target volumes are underestimated on a standard 3D-PET/CT scan when compared to target volumes defined on respiratory gated or 4D imaging [8-10]. This is primarily due to the effect of respiratory motion leading to count averaging across voxels through which the lesion moves or in which it resides for a relatively short period of the respiratory cycle.

When respiratory motion is taken into account the most common approach is to acquire a 4D-CT scan and this has become a routine clinical tool in some centres [11-13]. A recent study has found that adding a 4D-PET to a 4D-CT scan alters target volumes for lung tumours in approximately 23% of cases [14]. This would indicate that it may be important to incorporate both 4D-PET and 4D-CT information into the planning process.

The purpose of this study was therefore to investigate geographic miss of lung tumours due to respiratory motion for target volumes defined on a standard 3D-PET/CT scan when compared to target volumes defined on a 4D-PET/CT scan, assuming that the tumour should reside within the target volume for 100% of radiation delivery. In addition, we assessed the potential of applying individualised margins by exploring the degree of geographic miss when different margins are applied to the target volume to investigate the potential of individualised margins.

## Methods

### Patients

A total of 29 consecutive patients with staged for pulmonary malignancy who had completed both a 3D-PET/CT and 4D-PET/CT contemporaneously between August 2011 and May 2012. All patients gave their informed consent to participate in a study. Patients were included if they had an ^18^ F-fluoro-2′-deoxy-d-glucose (FDG) avid lung nodule with an SUV greater than 2.0 and larger than 15 mm in smallest diameter. These levels were chosen to minimise the effects of partial voluming and to ensure that the lesion could be resolved on the PET scan. This study was approved by the Clinical Research and Ethics Committee of the Peter MacCallum Cancer Centre.

### Scanning protocol

All patients were scanned on a GE Discovery 690 (GE Medical Systems Milwaukee, WI). The patients were scanned using the same acquisition protocol outlined in our previous work validating a 4D-PET maximum intensity projection (MIP) for target volume delineation [8]. The protocol involves first acquiring a standard whole-body PET/CT with the patient breathing freely. Then a single bed step 4D-PET/CT scan centred over the lesion of interest is immediately acquired after the standard PET/CT without the patient moving in between scans.

### Lesion contouring and planning

#### Gross Tumour Volume (GTV) definition

The lesion contouring protocol used was based on our published work that uses information from both the PET and CT scans to define the GTV [4]. All lung lesions were contoured by a single radiation oncologist experienced with this protocol. First, a 3D-GTV was defined on the standard whole-body PET/CT scan. Then a 4D-GTV was defined by the same observer on a 4D-PET/CT MIP. The 4D-PET/CT MIP consisted of a fused 4D-CT MIP and 4D-PET MIP as outlined in our previous work [8]. All contouring was performed on MIM Maestro imaging software (MIM 5.4.4, MIM Software Inc. Cleveland, OH, USA).

#### Planning Target Volume (PTV) definition

**Figure 1:**
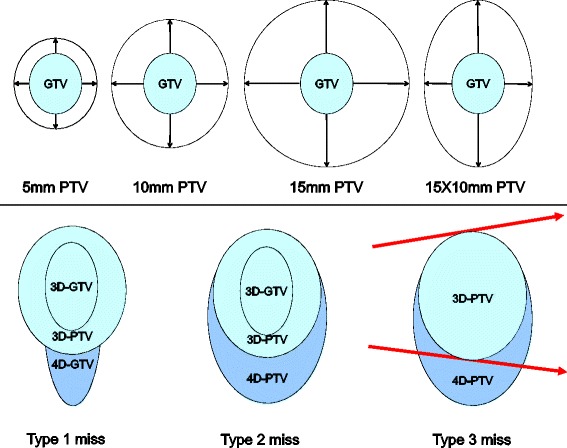
**The top row shows the four different margin expansions applied to the GTV to create four PTV contours.** The bottom row shows the types of geographic miss as a result of tumour motion due to respiration investigated.

Using the MIM contour expansion tool eight PTV’s were created, four 3D-PTV contours and four 4D-PTV contours. Firstly, three symmetrical PTV contours were created by expanding the 3D-GTV isotropically by 5 mm, 10 mm, and 15 mm. Subsequently, an asymmetrical contour was created by expanding the GTV anisotropically by 15 mm in the superior/inferior (SI) direction and 10 mm in the axial directions. Finally, these same four PTV’s (5 mm, 10 mm, 15 mm and anisotropic) were then created based on the 4D-GTV. This process is illustrated in Figure 1.

#### Treatment planning

All plans were generated on the XiO planning system (Computerized Medical Systems CMS, St Louis, MO, USA) using 6MV photons with 120 leaf millennium multileaf collimator (MLC) on a Varian machine. A 2.5 mm grid size was used and a fast superposition algorithm to account for tissue inhomogeneity. Each plan contained three or four fields arranged according to the tumour and critical organ location, prescribing 60Gy in 30Fractions consistent with institutional protocols and ICRU guidelines. All plans were calculated for 95% of the prescribed dose covered the PTV.

Each case had four plans developed, beginning with the 15 mm FB-PTV. The same beam arrangement was maintained for subsequent volumes, FB-10 mm, FB- 5 mm and Asymmetrical FB-PTV. The MLC arrangement was adjusted for the variation in volume size. The plans were transferred to the corresponding 4D - PTV and dosimetry was analysed using the dose volume histogram tool.

### Measurement of tumour motion

The tumour was first contoured on the 4D-CT scan in peak expiration phase when the tumour has the least amount of motion. Then the contour was automatically propagated across all the 4D-CT phases with deformable contour propagation [15]. The contours were manually checked for any gross errors introduced by the automatic process. This process copied a contour from one image to the next and applied a deformation to the contour to account for movement of an object. The motion of the tumour was measured by recording the distance the centroid position of the contour moved in the SI plane.

After contouring the patients were placed into 4 motion groups based on the amount of motion in the superior/inferior (SI) plane. The motion groups used were; < 5 mm, ≤ 10 mm, 10 ≤ 20 mm and greater than 20 mm. The tumour size was automatically measured in the superior/inferior plane by MIM. The ratio of the lesion motion to tumour size was then investigated for correlation between motion and miss. For example a 20 mm lesion that moved 20 mm would have a motion to size ratio of 1.0. If a 40 mm lesion motion moved 20 mm the ratio would be 0.5. This parameter takes into account both tumour size and motion.

### Analysis of geographic miss

Our previously published criteria were adapted to investigate geographic miss in 3D vs 4D defined target volumes [16]. 4D-PET/CT has been shown to account well for tumour motion it was considered the reference for which the 3D-PET/CT was compared against [17-19]. The types of geographic miss were defined as follows and are outlined in Figure 1.

- Type 1 miss – Any part of the 4D-GTV outside the 3D-PTV,
- Type 2 miss – Any part of the 4D-PTV outside the 3D-PTV,
- Type 3 miss – Any part of the 4D-PTV receiving less than 95% of the prescribed dose (where planning was based on the 3D-PTV).

The Type 3 miss was further divided into four groups:

- No Type 3 miss – 100% 4D-PTV receiving 95% of the prescribed dose,
- Minor Type 3 miss – >95% of 4D-PTV receiving 95% prescribed dose,
- Moderate Type 3 miss – 90-95% OF 4D-PTV receiving 95% prescribed dose,
- Significant Type 3 miss – <90% of the 4D-PTV receiving 95% prescribed dose.

### Data analysis

All statistical tests were carried out using GraphPad Prism 5 (GraphPad Software, LaJolla, Ca). The mean (±SD) 3D and 4D volumes were compared and tested for difference using a paired students’ t-test. For each type of miss the mean (±SD) volume that was missed was calculated. A chi-squared test was used to determine if there was a difference in the proportion of patients with a miss between the different PTV margin groups. Pearson’s correlation coefficients were used to examine the association between lesion motion and the volume of geographic miss as well as the ratio of size to motion and geographic miss. A p-value of < 0.05 was deemed a significant difference.

## Results

### Volume comparison

29 patients were included in the study with a mean age of 68 (range 45–87), 18 (62%) patients were male and 11 (38%) were female. A comparison of the 3D and 4D volumes is shown in Table 1. The 4D-GTV was on average 50% (range 2%-446%) larger than the 3D-GTV (p < 0.01). In turn the 4D-PTV’s were larger than the 3D-PTV’s across all margins applied (p < 0.01). The 10 mm 4D-PTV was on average 19% smaller than the 15 mm 3D-PTV (p < 0.01).

**Table 1:** **A comparison of mean 3D-PET/CT defined volumes to 4D-PET/CT defined volumes (total n = 29)**

	**3D volume**	**4D volume**		
	**(ml)**	**(ml)**	**Diff**	**p value**
GTV	15.7	23.5	50%	0.0013
PTV 5 mm	38.5	53.9	40%	0.0001
PTV 10 mm	72.8	96.1	32%	<0.0001
PTV 15 mm	119.2	155.9	31%	<0.0001
Asym	85.5	112.0	31%	<0.0001
15 mm vs 10 mm	119.2	96.1	19%	<0.0001

#### Type 1 miss - any part of the 4D-GTV outside the 3D-PTV

**Figure 2:**
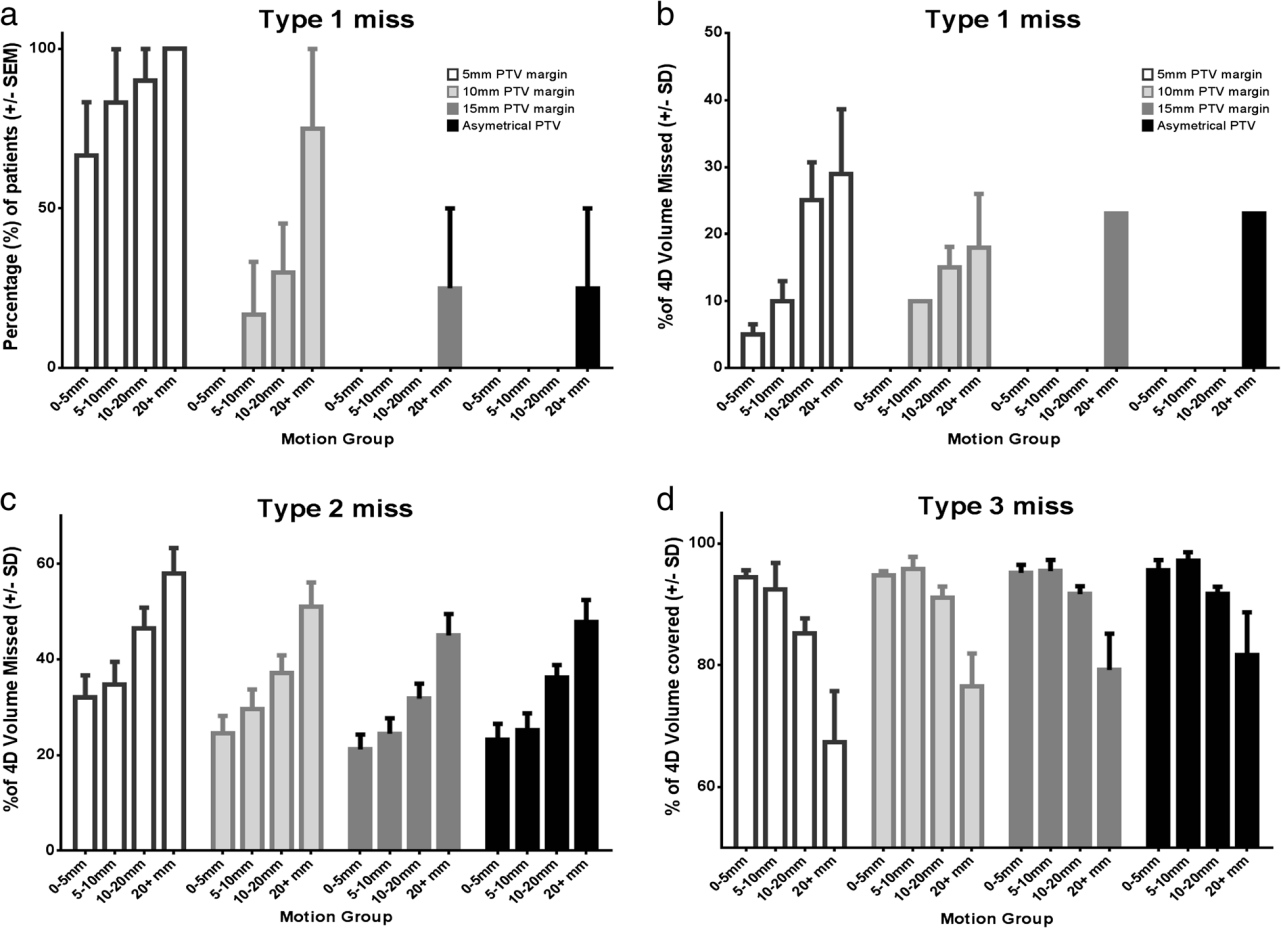
**Summary of results for the three types of geographic miss. a**: The proportions of patients with a type 1 miss in the four motion groups using the different PTV margins. **b**: The mean % of the 4D-GTV that was missed by the 3D-PTV when there was a type 1 miss. **c**: The mean % of the 4D-PTV that was missed by the 3D-PTV. **d**: The mean coverage of the 4D-PTV with 95% of the prescribed dose.

The proportions of patients with a Type 1 miss are shown in Figure 2a. When a standard 15 mm or asymmetrical PTV margin was used there was only one case of Type 1 miss in the group with the highest range of lesion motion. There was a significant difference in the proportion of patients with a Type 1 miss between the symmetrical PTV groups (15 mm vs 10 mm; p = 0.02, 10 mm vs 5 mm; chi-squared test, p < 0.01). There was no difference between the standard 15 mm PTV group and the asymmetrical PTV group.

**Figure 3:**
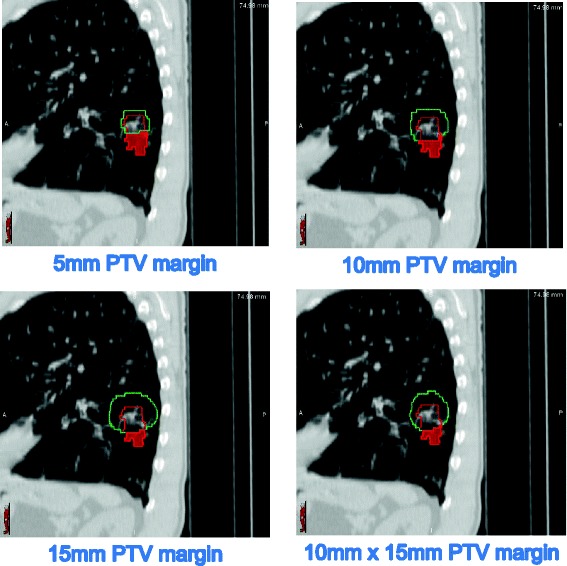
**CT scan of a patient with a small lower lobe tumour.** The 4D-GTV is shown in red and the 3D-PTV is shown in green. The highlighted red area is the part of the 4D-GTV that was not covered by the 3D-PTV for the margin applied.

As the lesion motion in the superior/inferior plane increased the proportion of patients with a Type 1 miss increased across all PTV margins (Figure 2a). Additionally, as the motion increased the percentage volume of the 4D-GTV that was missed by the 3D-PTV increased across all margins (Table 2b). An example of a patient with a type 1 miss using all four PTV margins is shown in Figure 3.

**Table 2:** **Type 3 miss: the severity of the Type 3 geographic miss in each of the motion groups based on the mean [±SD] coverage of the 4D-PTV with 95% of the prescribed dose (total n = 29)**

	**5 mm PTV severity**	**Mean 4D PTV coverage % [±SD]**	**10 mm PTV margin**	**Mean 4D PTV coverage % [±SD]**	**15 mm PTV margin**	**Mean 4D PTV coverage % [±SD]**	**Asymetrical PTV**	**Mean 4D PTV coverage % [±SD]**
**0- < 5 mm (n = 8)**	Minor	97 [2.9]	Minor	97 [1.7]	Minor	98 [3.3]	Minor	98 [4.2]
**5- < 10 mm (n = 6)**	Moderate	93 [5.9]	Moderate	95 [7.9]	Moderate	95 [4.8]	Minor	95 [5.4]
**10- < 20 mm (n = 11)**	Significant	86 [8.8]	Moderate	92 [5.3]	Moderate	92 [4.4]	Moderate	92 [3.9]
**20+ mm (n = 4)**	Significant	67 [16.7]	Significant	76 [10.9]	Significant	79 [12.0]	Significant	81 [14.0]

#### Type 2 miss - any part of the 4D-PTV outside the 3D-PTV

All patients for all PTV margins had a Type 2 geographic miss. The mean percentage of the 4D-PTV that was missed by the 3D-PTV is outlined in Figure 2c. A Pearson correlation showed a significant correlation (p < 0.01) between lesion motion and the amount of the 4D-PTV that was missed by the 3D-PTV across all PTV margins (15 mm r = 0.61, 10 mm r = 0.60, 5 mm r = 0.53, asym r = 0.60). A stronger correlation was found when the motion to lesion size ratio was used (15 mm r = 0.67, 10 mm r = 0.66, 5 mm r = 0.65, asym r = 0.68).

#### Type 3 miss - any part of the 4D-PTV receiving less than 95% of the prescribed dose (where planning was based on the 3D-PTV)

**Figure 4:**
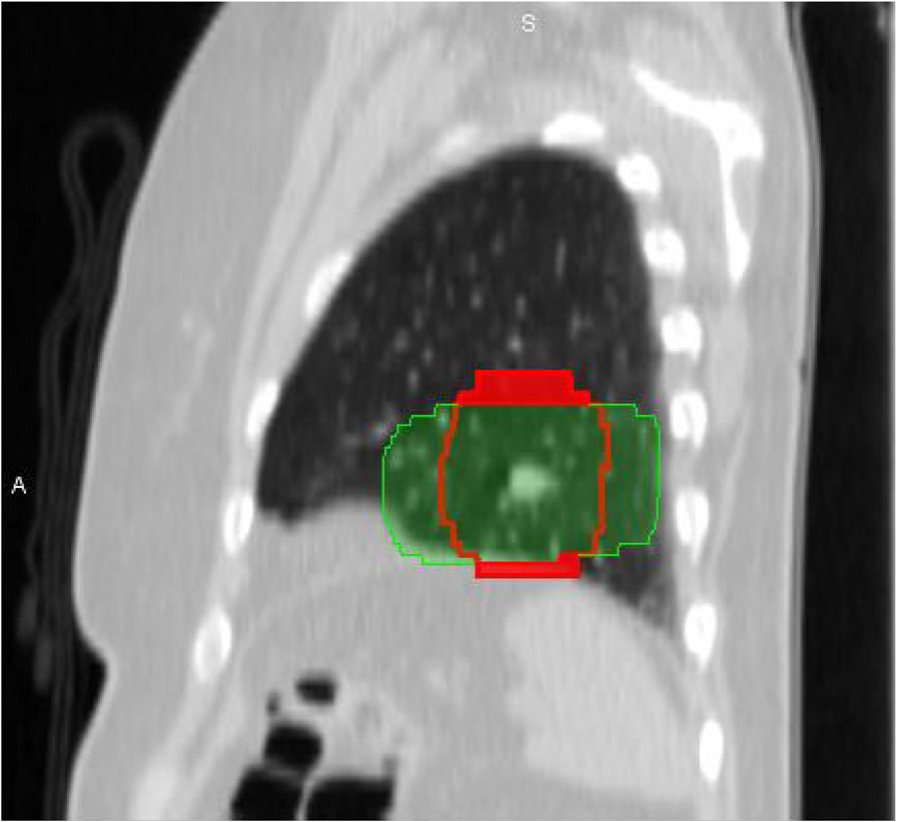
**A CT scan of a patient with a small lower lobe tumour.** The 4D-PTV is shown in red and the green area show the 95% isodose line. The solid red area is the part of the 4D-PTV that would not receive the prescribed dose and constitutes a Type 3 geographic miss.

There was a Type 3 miss in 25 out of 29 cases in the 5, 10, and 15 mm PTV margin groups. The asymmetrical margin had one additional Type 3 miss. The mean coverage of the 4D-PTV by the 3D-PTV plan is outlined in Figure 2d. A Pearson correlation showed an association (p < 0.01) between lesion motion and the percentage of the 4D-PTV that was missed by the 3D-PTV with all PTV margins (15 mm r = 0.57, 10 mm r = 0.54, 5 mm r = 0.57, asym r = 0.47). A stronger correlation was found when the motion to lesion size ratio was used (15 mm r = 0.61, 10 mm r = 0.60, 5 mm r = 0.64, asym r = 0.57). An example of a patient with a significant Type 3 miss is shown in Figure 4.

Table 2 outlines the data for the severity of the Type 3 miss based on the average 4D-PTV coverage. On average if there was less than 5 mm of motion any miss was minor (< 5%) and not likely to be clinically significant. When there was a high range of lesion motion (> 20 mm) the average target volume coverage resulted in a significant Type 3 miss. The mean coverage with 95% of the prescribed dose in the >20 mm group was; 5 mm = 67%, 10 mm = 76%, 15 mm = 79% and asym = 81%).

## Discussion

In this study we compared a standard technique of delineating the GTV on a 3D-PET/CT to an improved technique of 4D-PET/CT. Our work has found that 3D-PET/CT defined target volumes underestimate the true extent of respiratory motion despite being considered a ‘slow’ imaging modality that combines imaging information from all phases of the breathing cycle. This underestimation of the tumour motion led to increasingly severe geographic miss as the tumour motion increased. This is consistent with other studies comparing 3D and 4D-PET/CT volumes [8,9,18].

Results of this study also revealed that the ratio of lesion size to the amount of motion also appears to impact the severity of geographic miss. For example a small lesion that moves a large distance would likely have a worse geographic miss than a large lesion that moves across the same distance.

It has been possible for some time to perform both a 4D CT and a 4D PET scan in the same session on a PET/CT scanner [20]. While the technology has advanced and become more widely available it is still not commonly applied. One reason for this may be due to the limited number of studies showing clinical benefit that would compensate for the additional resources required to complete a 4D-PET/CT scan. The main reason to acquire a 4D scan to define target volumes is to minimise the risk of a geographic miss, thereby increasing the probability of attaining tumour control and enhanced patient outcomes. Sura and colleagues (2008) studied patterns of local failure in 34 lung lesions in 26 patients. They found that for a lung tumour receiving more than 60Gy the site of failure was mostly at the margins of the GTV [21]. As 3D-PET/CT can underestimate target volumes a possible explanation for the observed local failures is tumour motion due to normal respiration. A lung tumour that moves out of a defined target area may receive insufficient dose to areas that are missed, thereby increasing the risk of local failure.

Not surprisingly, these data show that the severity of the geographic miss rises significantly when a PTV margin is reduced from the standard 15 mm. This further confirms our institutional practice of using at least a 15 mm margin if there is no motion management in place. When employing highly conformal techniques such as stereotactic body radiotherapy (SABR) it is common to add only 5 mm margins to the GTV. If only a 3D-PET/CT scan or 3D-CT alone is used to delineate a GTV for stereotactic treatment these data suggests that there is a high likelihood of a significant geographic miss. The planning techniques are different in SABR with sharper dose gradients at the edge of the PTV and this may lead to and even more significant miss than described in this work.

In the delivery of radiation therapy to lung tumours there is always a need to reduce the dose to normal lung in order to minimise pulmonary toxicity. 4D-PET/CT imaging is not widely available so many centres may not be able to use this technique. Therefore in this study we compared geographic miss of a standard 15 mm margin to a 15x10mm asymmetrical margin. In this study rates of geographic miss were similar for symmetrical and asymmetrical margins. This was because most of the tumour motion occurs in the SI plane and reducing the margins in the other planes does not greatly increase the risk of a geographic miss. Using an asymmetric PTV margin is a method that could be employed on a 3D-PET/CT scan to reduce lung dose however further work in a larger cohort is warranted.

In most patients, if a 15 mm margin is applied to a 4D-GTV the resultant 4D-PTV will be significantly larger than a 3D-PTV, thereby potentially increasing normal lung dose. Conventional GTV to PTV margins allow for motion and set-up uncertainties. Assuming that the magnitude of both these uncertainties is similar and independent of each other our original margin of 15 mm on the 3D-GTV could be reduced. The use of 4D imaging provides not only the magnitude of motion but also defines the centre location more accurately than 3D scanning. We feel a margin reduction to 10 mm is justified in cases where motion has been accounted for in the GTV. As Table 1 shows this has the potential to reduce the actual irradiated volume significantly. The mean 4D-GTV with a 10 mm margin was 96.1 ml, which was significantly smaller than a 3D-GTV with a 15 mm margin (119.2 ml). However, individual radiotherapy departments would need to adjust safety margins individualised to department specific equipment and processes.

4D target volumes provide potential to customise a patients target volume and resultant radiotherapy plan based on their individual tumour motion [16]. What we and other authors have established that tumour motion is highly variable between patients and can be visualised using 4D scanning [22,23]. As it is becoming common practice to use a PET/CT in the planning process it would be an efficient use of resources to add a 4D-PET/CT at this time point. Depending on the amount of tumour motion observed a target volume could be tailored to suit their individual lesion trajectory. This will either ensure correct tumour coverage for a highly mobile tumour or allow normal tissue dose reduction in fixed tumours. Conversely these data show that if a radiotherapy department does not have any motion management equipment with their PET/CT scanner then they should not attempt to reduce safety margins from the larger 15 mm expansion. Further work investigating personalising target volumes is warranted and may improve local tumour control or reduce risks of side effects.

Based on our retrospective study there is no way to know if the types of geographic miss measured translated to decreases in tumour control. However it is known that the radiation dose to a tumour is a good predictor for local control (*18, 19*). Also a geographic miss such as described in this study would constitute a protocol violation and as was shown by Peters et al. adherence to protocol is a good predictor of overall survival [24]. It is therefore reasonable to infer that any form of geographic miss would lead to a reduction in tumour dose and subsequently increase the risk of treatment failure. A 4D-PET/CT scan incorporated into a planning PET/CT is an achievable method of accurately establishing and accounting for the adverse effects of respiratory motion on treatment success.

## Conclusions

Without any form of motion suppression the current standard of a 3D- PET/CT and 15 mm PTV margin employed for lung lesions has an increasing risk of significant geographic miss in particular when tumour motion increases. Use of smaller asymmetric margins in the cranio-caudal direction does not comprise tumour coverage. Reducing PTV margins for volumes defined on 3D-PET/CT will greatly increase the chance and severity of a geometric miss due to respiratory motion. 4D-imaging reduces the risk of geographic miss across the population of tumour sizes and magnitude of motion investigated in the study.
